# Self-reported Mental and Physical Health Among Norwegian Adolescents Before and During the COVID-19 Pandemic

**DOI:** 10.1001/jamanetworkopen.2021.21934

**Published:** 2021-08-24

**Authors:** Jasmina Burdzovic Andreas, Geir Scott Brunborg

**Affiliations:** 1 Department of Alcohol, Tobacco, and Drugs, Norwegian Institute of Public Health, Oslo, Norway; 2 Department. of Psychology, University of Oslo, Oslo, Norway

## Abstract

**Importance:**

The COVID-19 pandemic and resulting conditions may negatively affect adolescents.

**Objective:**

To examine aspects of self-reported mental and physical health among adolescents in Norway before and during the pandemic, including the role of pandemic-associated anxiety.

**Design, Setting, and Participants:**

This cohort study examined a diverse nationwide sample of grade 11 students from the longitudinal MyLife study in Norway. The original study recruitment of all 8th, 9th, and 10th graders from the same middle schools facilitated identification of 2 sociodemographically comparable cohorts assessed in October to December 2018 and 2019, before the COVID-19 pandemic, and October to December 2020, during the pandemic. School entry and enrollment in Norway is determined by the birth year, and students usually start high school (11th grade) during the fall of the year of their 16th birthday. Data were analyzed from March to June 2021.

**Exposures:**

The COVID-19 pandemic and associated conditions in Norway.

**Main Outcomes and Measures:**

In grades 10 and 11, adolescents reported their depression symptoms using the Patient Health Questionnaire-9 (cutoff scores for moderate/severe depression, ≥15), number of close friends, physical health, and organized sports participation. Cohort differences were examined with a set of nested regression models, incrementally controlling for sociodemographic covariates and grade 10 outcomes.

**Results:**

A sample of 2536 adolescents (1505 [59.4%] girls) was analyzed, including 1621 adolescents before the pandemic and 915 adolescents during the pandemic, of whom 158 adolescents (17.3%) reported high pandemic anxiety. The only significant difference in outcomes between the COVID-19 cohort and the pre–COVID-19 cohort were lower odds of organized sports participation (adjusted odds ratio [aOR], 0.69; 95% CI, 0.56-0.87). However, in subanalyses comparing adolescents with high anxiety during the COVID-19 pandemic with adolescents in the pre–COVID-19 cohort, adolescents with high pandemic anxiety were more likely to experience clinical-level depression symptoms (aOR, 2.17; 95% CI, 1.39-3.37) and poor physical health (aOR, 1.53; 95% CI, 1.01-2.31).

**Conclusions and Relevance:**

In this cohort study of Norwegian adolescents, adolescents who started high school during the pandemic year had lower odds of organized sports participation in late 2020, but were otherwise comparable in terms of self-reported mental and physical health with their pre–COVID-19 counterparts. However, adolescents in the COVID-19 cohort experiencing high pandemic-related anxiety had significantly greater odds of poorer mental and physical health than adolescents in the pre–COVID-19 cohort. Strategies aiming to mitigate the impact of COVID-19 may benefit from identifying youth disproportionally affected by the pandemic conditions.

## Key Points

**Question:**

Are there differences in self-reported depression symptoms, friendships, physical health, and organized sports participation among adolescents in Norway before vs during the COVID-19 pandemic and across levels of experienced pandemic-related anxiety?

**Findings:**

This cohort study including 2536 adolescents found that Norwegian adolescents starting high school during the COVID-19-year had lower odds of sports participation than their peers starting high school in preceding years, but no significant differences in depression symptoms, friendships, and physical health. However, elevated depression symptoms and poor physical health were significantly more common in the subgroup of adolescents experiencing high pandemic-related anxiety in the COVID-19 cohort compared with their peers in the pre–COVID cohort.

**Meaning:**

These findings suggest that most adolescents from this sample coped adequately with the pandemic conditions but that strategies aiming to mitigate the impact of COVID-19 may benefit from identifying youth disproportionally affected by the pandemic-related anxieties.

## Teaser

This cohort compares aspects of self-reported mental and physical health among adolescents in Norway before and during the COVID-19 pandemic.

## Introduction

The emergence of COVID-19 in late 2019 and the subsequent containment measures beginning in 2020 profoundly disrupted the lives of children and families around the world. Norway, a sparsely populated Nordic country currently ranked number 1 in the Human Development Index,^1^ was the first country to implement a nationwide lockdown on March 12, 2020, after precipitous increases in COVID-19 cases indicative of community transmission. The lockdown was accompanied by several comprehensive economic packages,^2,3^ and it included mandatory physical closures of all educational institutions, from preschools to universities; recommendations for working from home whenever possible; limitations on nonessential services, such that only grocery stores, pharmacies, and gas stations remained open; orders for self-isolation and quarantine in cases of exposure or infection; regulations and prohibitions of social, cultural, and religious gatherings; cancellations of organized sports activities and competitions; and restrictions of nonessential travel, such that nonresidents were essentially barred from entering the country and residents were prohibited from staying overnight in their cabins across municipal borders.^2,3,4,5,6,7^ Such restrictions on autonomy, sports engagements, and freedom of movement were considered “draconian” by some^3^ in a society with high cultural commitment to youth sports (eg, >90% of adolescents in Norway join a sports club) and national identification with holiday travel and cabin pastime (eg, 4 of 10 households in Norway have access to a country cabin).^8,9,10,11^

This initial set of suppression strategies was modified on an as-needed basis throughout 2020, gradually reopening the society and easing into control strategies reflecting regional conditions.^3,6^ For example, additional directives (eg, face masks on public transport) were introduced later in many regions. Although all schools were allowed to reopen on May 11, 2020, 2 months after the initial closures, the actual instruction remained subject to continued disruption and adjustment following the traffic model of the local red, yellow, or green emergency conditions reflecting community spread.^6,12^ Similarly, sports arrangements and facilities were allowed to resume in May and June, conditional on firm adherence to the control measures,^6^ but were in reality operating far from standard conditions. Another set of stricter national policies was implemented on October 26, 2020, after the spike in hospitalizations following summer holidays.^6^ Ultimately, Norway did not register elevated mortality during the first pandemic year,^3,13^ yet all aspects of society were deeply affected.^3,4,5,7^

There is an overarching agreement that these extraordinary circumstances may contribute to a global public mental health crisis.^14,15^ Children’s and adolescents’ well-being remains a particular concern,^16,17,18,19,20^ as their mental and physical health may have been shaped by the pandemic conditions above and beyond the infection risk. Emerging evidence suggests that there is a negative association between the pandemic and multiple domains of young people’s health and well-being, especially mental and social health (eg, depression, quality of life, loneliness)^17,21,22,23,24,25,26,27,28^ and physical activity.^29,30,31,32^ Consequently, understanding in what ways and to what extent the pandemic is associated with health and well-being outcomes among various youth subpopulations remains a public health priority, with the related need for high-quality, nuanced research.^18,20,33^ Such research could also help clarify what strategies may be needed to mitigate the impact of COVID-19 and accompanying policies, and identify the groups in greatest need of such strategies.

This study addresses such public health needs and research gaps by examining multiple aspects of adolescent self-reported mental (ie, depression symptoms and close friendships) and physical (ie, physical health and participation in organized sports) health using nationwide prospective cohorts of Norwegian adolescents from before and during the pandemic. We additionally focused on youth who may have experienced high pandemic-related anxiety, stress, or burden, as such groups may be at particularly high risk for adverse outcomes.^5,17,34,35^

## Methods

This cohort study was approved by the Norwegian Data Protection Authority (DPA) after ethical evaluation by the National Committee for Research Ethics in the Social Sciences and the Humanities. Written parental consent was obtained for all participants younger than 16 years before baseline data collection; students provided their assent through participation. This study is reported following the Strengthening the Reporting of Observational Studies in Epidemiology (STROBE) reporting guideline.

### Study Design and Procedures

The ongoing MyLife study^36^ provided a unique opportunity to isolate and quantify the putative risk of the COVID-19 pandemic on adolescent mental and physical health by comparing students entering high school in 2020 with a sociodemographically comparable cohort entering high school in the 2 preceding years. The MyLife study aimed to recruit entire cohorts of 8th, 9th, and 10th grade students from 33 middle schools throughout Norway. All eligible students were invited to complete annual electronic surveys during class time while in middle school (ie, grades 8-10) and individually once in high school (ie, grades 11-13). Baseline data collection took place during the 2017 fall semester. So far, there have been 3 follow-ups; T2 in 2018, T3 in 2019, and T4 in 2020. Detailed study protocol and core cohort descriptions are provided elsewhere.^36^

The accelerated longitudinal design^37^ made it possible to desegregate data collection waves, school grades, and the secular periods of interest (ie, before vs during the pandemic). Grade 11 was selected for all comparisons because our youngest baseline cohort entered high school (ie, grade 11) in August 2020. Because school entry and enrollment in Norway is determined by the birth year, the variation in age is low, and students usually start high school during the fall of the year of their 16th birthday. Because all students were assessed in grades 10 and 11 by 2020, this approach also provided the most robust analytical sample and within-person control for previous levels of all examined outcomes.

As the T4 data collection took place during the 2020 fall semester (ie, October-December), our COVID-19 cohort of 11th graders had experienced approximately 8 to 10 months of the pandemic and the related suppression and containment measures in Norway at that time.

### Sample and Measures

We analyzed 2 sociodemographically comparable cohorts. One cohort included students entering high school in 2020, ie, the COVID-19 cohort. Students entering high school in 2019 and 2018 were combined into the single pre–COVID-19 cohort for ease of analyses.

### Exposures

The COVID-19 pandemic and the associated containment policies in Norway,^2,3^ as captured by 2 contiguous cohorts of grade 11 students, were the key exposures of substantive interest. During the 2020 assessment, students responded to the 3 newly added items based on the Pandemic Anxiety Scale^34^ about how worried they were about the possible SARS-CoV-2 infections and the remote schooling situation. The original responses (from 1 indicating not worried at all to 3, worried a lot) were calculated as means; scores greater than 2 were considered high pandemic anxiety (HPA). Similar short ad hoc measures were used in other recent studies^5,26,27,28^ to assess pandemic-related concerns among youth.

### Outcomes

Students completed comprehensive surveys during the fall semesters of grades 10 and 11. We examined several aspects of adolescent mental (ie, depression symptoms and friendships) and physical (ie, physical health and participation in organized sports) health, as reported by adolescents themselves.

#### Depression Symptoms

Students completed the 9-item Patient Health Questionnaire (PHQ-9; adolescent version),^38^ which has been used successfully in several COVID-19 studies^7,21,35,39^ and in Norwegian adolescent and adult samples.^7,40,41^ The scale demonstrated solid psychometric properties in this study (Cronbach α ≥ .90 at each assessment and at each grade). The PHQ-9 items measure depressive symptoms during the past 14 days using a 4-point scale ranging from 0, indicating not at all, to 3, almost every day, thus generating 0 to 27 sum scale scores. PHQ-9 scores of 15 or greater are indicative of moderately severe to severe depression and closely align with the *Diagnostic and Statistical Manual of Mental Disorders* (Fourth Edition) (DSM-IV)^42^ diagnostic criteria for major depressive disorder.^38^

#### Friendships

Adolescents reported how many friends they considered close and trustworthy. The original responses of none, and not sure (if any) were collapsed into 0, and other responses were 1, 2, or 3 or more.

#### Physical Health

Adolescents evaluated their physical health during the past 12 months using response options from 1, indicating very good, to 5, very bad. These responses were dichotomized to reflect poor physical health (ie, responses of bad or very bad) vs everyone else.

#### Organized Sports Participation

Adolescents reported how often they participated in organized sports (eg, soccer, swimming) during the past 30 days. The original responses were dichotomized into not at all vs at least once.

### Covariates

Both cohorts were recruited from the same middle schools; thus, they were sociodemographically comparable in all aspects except for the COVID-19 historical period. Nevertheless, we accounted for possible individual-level differences and characteristics.

### Sociodemographic Characteristics

At study baseline, adolescents reported their sex, parental cohabitation, and the language spoken at home (a proxy for immigrant background). In addition, adolescents responded to the adolescent version of the MacArthur Scale of Subjective Social Status,^43^ in which scores of 1 reflected the lowest and scores of 10 reflected the highest subjective ranking across neighborhood families. The mean values of 2017 and 2018 reports were used and categorized to reflect 3 levels of subjective social status: low (score, ≤4.5), middle (score, 4.5-7.5), and high (score, 8-10).

### Negative Life Events

Adolescents selected whether they experienced any of 15 major negative life events in the past year,^44^ such as the serious illness or death of a close family member, relocation, financial problems, and breakup of close relationships. The responses were summed up to obtain the Negative Life Events Index at grade 11.

### Statistical Analysis

Missing values on all covariates were classified into the dummy unknown category and were included as such in all models; however, one adolescent without any data on the pandemic anxiety items was excluded from subanalyses. Cohort comparisons provided tests of the COVID-19 excess risk. All outcomes were examined using linear (for continuous variables), logistic (for binary variables), and Poisson (for count variables) regression models. Specifically, we fit a set of nested models for each outcome in which we first estimated a crude model (model 0); then a model adjusted for adolescent sex, parental cohabitation, subjective social status, immigrant background, and grade 11 Negative Life Events Index (model 1); and finally a model adjusted for all aforementioned covariates and earlier levels of the examined outcome (model 2). An identical set of models was estimated across the pre–COVID-19 cohort and COVID-19 low pandemic anxiety and COVID-19 HPA subgroups to examine the putative association of the experienced pandemic anxiety with outcomes.

Finally, we performed a set of sensitivity analyses to address nonparticipation during grades 10 and 11. A total of 360 adolescents who did not complete grade 10 surveys returned at grade 11, resulting in reduced analytical sample sizes in the fully adjusted model 2 for all outcomes. Thus, we reran model 2 in which we recoded the respective grade 10 covariates to reflect 3 basic conditions: outcome present (eg, PHQ-9 scores within clinical range), outcome absent (eg, PHQ-9 scores within reference range), and outcome unknown.

All analyses were conducted in Stata statistical software version 15 (StataCorp). Robust SEs were estimated with the *vce* (cluster) option,^45^ which accounted for the school-level nesting resulting from the original school-based sampling. All reported probabilities, means, and counts were estimated at the mean values of the remaining covariates using the -margins command. *P* values were 2-sided, and statistical significance was set at .05. Data were analyzed from March to June 2021.

## Results

### Sample Characteristics

From the core sample of 3512 eligible adolescents, 2975 adolescents (1505 [59.4%] girls) had valid data at grade 11 assessment. This included 1621 adolescents assessed before the pandemic and 915 adolescents assessed during the pandemic. The 2 examined cohorts were comparable on most sociodemographic characteristics (Table 1). HPA was reported by 158 adolescents (17.3%) in the COVID-19 cohort.

**Table 1.** Characteristics of Participating Adolescents

Characteristic	No. (%) (N = 2536)^a^			
	Pre–COVID-19 cohort (n = 1621)	COVID-19 cohort^b^		
		All (n = 915)	LPA (n = 756)	HPA (n = 158)
Sex				
Girls	952 (58.7)	553 (60.4)	430 (56.9)	123 (77.8)
Boys	669 (41.3)	361 (59.6)	326 (43.1)	35 (22.2)
Parental cohabitation				
Yes	988 (60.9)	609 (66.5)	503 (66.5)	105 (66.4)
No	384 (23.7)	212 (23.2)	172 (22.7)	40 (25.3)
Unknown	249 (15.4)	94 (10.3)	81 (10.7)	13 (8.2)
Subjective social status				
Low	59 (3.6)	24 (2.6)	18 (2.4)	6 (3.8)
Average	857 (52.8)	492 (53.8)	399 (52.8)	93 (58.8)
High	646 (39.9)	354 (38.7)	298 (39.4)	55 (34.8)
Unknown	59 (3.6)	45 (4.9)	41 (5.4)	4 (2.5)
Immigrant background				
No	1210 (74.7)	716 (78.2)	589 (77.9)	126 (79.8)
Yes	164 (10.2)	103 (11.2)	84 (11.1)	19 (12.0)
Unknown	247 (15.2)	96 (10.5)	83 (11.0)	13 (8.2)
NLE Index, mean (SD)	1.65 (1.82)	1.74 (1.89)	1.58 (1.81)	2.53 (2.09)

Abbreviations: HPA, high pandemic anxiety; LPA, low pandemic anxiety; NLE, negative life events.

^a^ All covariates were assessed at baseline, except for the Subjective Social Status, which was assessed as the mean of the first and second assessments, and NLE Index, which was assessed at the beginning of grade 11.

^b^ Scores greater than 2 on the 3-item Pandemic Anxiety Index differentiated adolescents in the COVID-19 cohort with HPA from those with LPA.

### Adolescent Mental and Physical Health as a Function of Pandemic Conditions

Table 2 presents the regression estimates from models 0 through 2 for all examined outcomes. The only statistically significant difference was lower odds of organized sports participation in the COVID-19 cohort compared with the pre–COVID-19 cohort. Specifically, approximately 3 in 4 adolescents (75.3%; 95% CI, 71.1%-79.4%) in the COVID-19 cohort participated in organized sports during the past month, compared with 4 in 5 adolescents (81.4%; 95% CI, 78.1%-84.7%) in the pre–COVID-19 cohort (fully adjusted model 2: adjusted odds ratio [aOR], 0.69; 95% CI, 0.56-0.87).

**Table 2.** Self-reported Mental and Physical Health in Grade 11 Student Cohorts From Before and During the COVID-19 Pandemic

Outcome	Pre–COVID-19 cohort	COVID-19 cohort	Estimate	*P* value
PHQ-9, mean (95% CI)^a^				
Model 0^b^	8.17 (7.76-8.57)	8.58 (8.10-9.07)	0.42 (–0.07 to 0.90)^c^	.09
Model 1^d^	8.22 (7.84-8.61)	8.48 (8.08-8.88)	0.26 (–0.08 to .59)^c^	.13
Model 2^e^	8.14 (7.90-8.38)	8.29 (7.88-8.67)	0.15 (–0.19 to 0.49)^c^	.38
PHQ-9: clinical-range, % (95% CI)^a^				
Model 0^b^	14.29 (12.25-16.33)	17.07 (5.07-19.07)	1.23 (0.99 to 1.52)^f^	.05
Model 1^d^	10.68 (8.56-12.80)	12.69 (10.75-14.64)	1.21 (0.97 to 1.52)^f^	.08
Model 2^e^	9.17 (7.09-11.24)	9.33 (7.17-11.49)	1.02 (0.76 to 1.37)^f^	.89
Friendships^,^, mean (95% CI)^g^				
Model 0^b^	2.24 (2.18-2.29)	2.14 (2.05-2.24)	0.96 (0.91 to 1.01)^h^	.08
Model 1^d^	2.22 (2.17-2.27)	2.14 (2.05-2.23)	0.96 (0.92 to 1.01)^h^	.11
Model 2^e^	2.21 (2.17-2.26)	2.14 (2.07-2.22)	0.97 (0.93 to 1.01)^h^	.10
Poor physical health, % (95% CI)^i^				
Model 0^b^	31.62 (28.74-34.50)	32.97 (28.95-36.98)	1.06 (0.88 to 1.28)^f^	.52
Model 1^d^	30.63 (27.98-33.28)	31.23 (27.46-35.00)	1.03 (0.85 to 1.24)^f^	.77
Model 2^e^	27.31 (24.68-29.93)	27.57 (24.21-30.93)	1.01 (0.82 to 1.24)^f^	.90
Organized sports, % (95% CI)^j^				
Model 0^b^	76.86 (72.78-80.95)	73.62 (69.32-77.93)	0.84 (0.71 to 0.99)^f^	.04
Model 1^d^	77.50 (73.70-81.30)	74.33 (70.14-78.52)	0.84 (0.71 to 0.99)^f^	.04
Model 2^e^	81.42 (78.10-84.74)	75.27 (71.15-79.39)	0.69 (0.55 to 0.86)^f^	.001

Abbreviation: PHQ-9, 9-item Patient Health Questionnaire.

^a^ Possible range of scores is 0 to 27. PHQ-9 scores 15 or higher closely align with the Diagnostic and Statistical Manual of Mental Disorders (Fourth Edition)^42^ diagnostic criteria for major depressive disorder.^38^

^b^ Model 0 is unadjusted.

^c^ Expressed as unstandardized regression coefficient estimates (95% CI).

^d^ Model 1 is adjusted for all covariates.

^e^ Model 2 is adjusted for all covariates and grade 10 outcomes.

^f^ Expressed as odds ratios (95% CIs).

^g^ Number of close and trusted friends reported by adolescents (possible range, 0-3).

^h^ Expressed as incidence risk ratios (95% CIs).

^i^ Proportion of adolescents self-evaluating their physical health as bad or very bad.

^j^ Proportion of adolescents reporting participation in organized sports at least once in the past month.

### Adolescent Mental and Physical Health as a Function of Pandemic Conditions and Associated Anxiety

Table 3 presents the regression estimates from models 0 through 2 while considering the role of pandemic anxiety in the COVID-19 cohort for all outcomes. In addition to the already established lower odds of organized sports participation, both clinical-level depression symptoms (aOR, 2.17; 95% CI, 1.39-3.30) and poor physical health (aOR, 1.53; 95% CI, 1.01-2.31) were significantly more common in the HPA group from the COVID-19 cohort than in the Pre–COVID-19 cohort.

**Table 3.** Self-reported Mental and Physical Health in Grade 11 Student Cohorts From Before and During the COVID-19 Pandemic Across Reported Pandemic Anxiety

Outcome	Pre–COVID-19 cohort	LPA COVID-19 cohort^a^	HPA COVID-19 cohort^a^	Pre–COVID-19 vs LPA COVID-19		Pre–COVID-19 vs HPA COVID-19	
				Estimate	*P* value	Estimate	*P* value
PHQ-9, mean (95% CI)^b^							
Model 0^c^	8.17 (7.8-8.6)	7.92 (7.4-8.4)	11.76 (10.7-12.8)	–0.24 (–0.74 to 0.24)^d^	.32	3.59 (2.48 to 4.70)^d^	<.001
Model 1^e^	8.22 (7.8-8.6)	8.15 (7.7-8.6)	10.09 (9.1-11.1)	–0.07 (–0.37 to 0.22)^d^	.61	1.86 (0.82 to 2.90)^d^	.001
Model 2^f^	8.13 (7.9-8.4)	8.00 (7.5-8.5)	9.50 (8.7-10.3)	–0.13 (–0.53 to 0.27)^d^	.51	1.37 (0.50 to 2.23)^d^	.003
PHQ-9 Clinical-range, % (95% CI)^b^							
Model 0^c^	14.3 (12.3-16.3)	13.5 (11.0-15.9)	34.2 (28.2-40.1)	0.93 (0.72 to 1.19)^g^	.58	3.11 (2.21 to 4.38)^g^	<.001
Model 1^e^	10.8 (8.7-12.9)	10.7 (8.4-12.9)	21.1 (15.9-26.3)	0.98 (0.76 to 1.28)^g^	.92	2.21 (1.53 to 3.17)^g^	<.001
Model 2^f^	9.3 (7.2-11.4)	7.0 (4.6-9.4)	18.2 (12.3-24.1)	0.74 (0.50 to 1.08)^g^	.12	2.16 (1.39 to 3.37)^g^	.001
Friendships, mean (95% CI)^h^							
Model 0^c^	2.24 (2.2-2.3)	2.14 (2.0-2.2)	2.15 (1.9-2.3)	0.96 (0.91 to 1.01)^i^	.08	0.96 (0.87 to 1.05)^i^	.37
Model 1^e^	2.22 (2.2-2.3)	2.13 (2.0-2.2)	2.18 (2.0-2.4)	0.96 (0.91 to 1.01)^i^	.09	0.98 (0.89 to 1.07)^i^	.62
Model 2^f^	2.21 (2.2-2.3)	2.15 (2.1-2.2)	2.13 (1.9-2.3)	0.97 (0.93 to 1.01)^i^	.19	0.96 (0.89 to 1.04)^i^	.31
Poor physical health, % (95% CI)^j^							
Model 0^c^	31.6 (28.7-34.5)	30.6 (26.7-34.5)	44.3 (37.5-51.1)	0.96 (0.79 to 1.15)^g^	.64	1.72 (1.31 to 2.25)^g^	<.001
Model 1^e^	30.7 (28.0-33.3)	30.0 (26.3-33.6)	37.4 (30.2-44.5)	0.97 (0.79 to 1.17)^g^	.73	1.35 (1.00 to 1.81)^g^	.049
Model 2^f^	27.4 (24.7-30.0)	25.6 (22.1-29.1)	35.6 (27.4-45.7)	0.91 (0.73 to 1.14)^g^	.43	1.53 (1.01 to 2.31)^g^	.04
Organized sports, % (95% CI)^k^							
Model 0^c^	76.8 (72.8-80.9)	74.7 (69.9-79.5)	68.4 (62.3-74.3)	0.89 (0.73 to 1.08)^g^	.24	0.65 (0.49 to 0.86)^g^	.002
Model 1^e^	77.5 (73.7-81.3)	75.2 (70.5-79.8)	70.3 (64.5-76.1)	0.88 (0.72 to 1.08)^g^	.22	0.69 (0.53 to 0.89)^g^	.005
Model 2^f^	81.4 (78.1-84.7)	76.2 (71.4-81.0)	71.1 (63.8-78.4)	0.73 (0.55 to 0.96)^g^	.02	0.56 (0.39 to 0.80)^g^	.001

Abbreviations: PHQ-9, 9-item Patient Health Questionnaire; HPA, high pandemic anxiety; LPA, low pandemic anxiety.

^a^ Scores greater than 2 on the 3-item Pandemic Anxiety Index differentiated HPA vs LPA subgroups in the COVID-19 cohort.

^b^ Possible range of scores is 0 to 27. PHQ-9 scores 15 or higher closely align with the Diagnostic and Statistical Manual of Mental Disorders (Fourth Edition)^42^ diagnostic criteria for major depressive disorder.^38^

^c^ Model 0 is unadjusted.

^d^ Expressed as unstandardized regression coefficient estimates (95% CI).

^e^ Model 1 is adjusted for all covariates.

^f^ Model 2 is adjusted for all covariates and grade 10 outcomes. Because not all adolescents had grade 10 data available, the group sizes for model 2 analyses were 1398 adolescents in the pre–COVID-19 cohort, 637 adolescents in the LPA COVID-19 subgroup, and 140 adolescents in the HPA COVID-19 subgroup.

^g^ Expressed as odds ratios (95% CIs).

^h^ Number of close and trusted friends reported by adolescents (possible range, 0-3).

^i^ Expressed as incidence risk ratios (95% CIs).

^j^ Proportion of adolescents self-evaluating their physical health as bad or very bad.

^k^ Proportion of adolescents reporting participation in organized sports at least once in the past month.

### Sensitivity Analyses

The main model 2 analyses for key outcomes were repeated to account for grade 10 nonparticipation, such that the grade 10 indicators reflected 3 basic conditions: outcome present, outcome absent, and outcome unknown. The results from these analyses were conceptually congruent with the model 2 results reported in Table 2, suggesting that differences in participation in grades 10 to 11 were not systematic.

## Discussion

To our knowledge, this cohort study is the first study to examine multiple aspects of mental and physical health among adolescents entering high school in Norway—specifically, depression symptoms, friendship, physical health, and organized sports participation—in association with COVID-19 pandemic conditions. We capitalized on an ongoing accelerated longitudinal design study during the specific secular period defined by a global pandemic, ie, during a large-scale experiment by nature.^46,47^ Because the examined grade 11 cohorts were recruited from the same schools throughout Norway and were sociodemographically comparable except for the assessment calendar year (ie, pre–COVID-19 vs during COVID-19), the analyses were adjusted for a range of sociodemographic covariates, and prior levels of the examined outcomes were accounted for at the individual level, the observed differences can be understood to be primarily associated with the COVID-19 conditions, including the resulting anxiety.

The estimates from the fully adjusted models show that adolescents from our sample were able to cope adequately with the pandemic conditions by late 2020. This may be owing to multiple structural and individual factors, ranging from a trusted and well-funded welfare state and its thus far successful pandemic management^2,3^ to youth in Norway being well-adjusted with strong family support before the pandemic onset.^48^ Further research is needed to illuminate putative stress-buffering and resilience-promoting processes.^49,50^

Outcomes associated with the pandemic were evident only in the lower odds of organized sports participation in the COVID-19 cohort. Considering that most adolescents in Norway belong to a sports club,^9^ these findings may be directly linked to the pandemic suppression and control measures imposed on local sports clubs. They also reiterate growing concerns about physical activity in the context of pandemic-related facility closures and event cancellations.^29,30,32^ They are also greatly relevant in a society characterized by both high cultural commitment to sports and high adolescent sports participation,^9,10^ especially during the pandemic conditions, when physical activity may provide beneficial coping strategies^51^ and promote resilience.^52^

We also considered the role of pandemic-related anxiety and stress, and found that the depression symptoms and poor physical health were significantly more common in the COVID-19 subgroup experiencing HPA than in the pre–COVID-19 cohort. This subgroup, characterized by elevated pandemic-related anxiety, may have borne the brunt of the pandemic burden, as evidenced in poorer self-reported mental and physical health. Therefore, closer attention to youth experiencing disproportionate pandemic-related anxiety and, possibly, a genuine pandemic-related burden, is warranted. Further research is needed to understand the characteristics and mechanisms placing these adolescents at greater risk of HPA and compromised health outcomes.

### Limitations

This study has some limitations. Our results should be understood within the temporal, social, and political context of the COVID-19 pandemic in Norway,^2,3^ as well as within the limitations of the sample. First, at the time of our study, Norway had experienced no excess mortality and only a relatively moderate economic hardship owing to a set of well-funded governmental policies.^2,3,13^ Second, our 2020 data collection took place approximately 8 to 10 months into the pandemic, during the peak of the second wave but after the school summer vacations and before the winter season. How the examined outcomes developed after the prolonged and more sustained pandemic exposure (ie, until spring 2021 or later) is not known from these relatively short-term data. Nevertheless, our late 2020 assessment extends the timeline considerably beyond the initial pandemic periods examined in other Norwegian studies.^4,5,28^

Third, although we examined a nationwide and sociodemographically diverse sample, it is not clear how representative this sample may be, given the omission of the nation’s capital, Oslo, which was most severely affected by the pandemic and, consequently, by the implemented suppression and control strategies. For example, other Norwegian reports examining youth adjustment under the pandemic conditions have noted significant decreases in life satisfaction among adolescents in Oslo^28^ but improvements in everyday life among adolescents in Bergen^5^ and only age-associated increases in depression in a representative sample of adolescents.^4^ It is possible that these regional variations actually reflected the regional infection rates, corresponding local measures, and the resulting pandemic anxiety as shown in our results. Fourth, we used rather conservative cutoff values for the PHQ-9 depression screener and somewhat ad hoc measures for pandemic-related anxiety. Additionally, it is possible that some adolescents substituted their organized sports participation with more unstructured exercise routines during the pandemic period, but it is not known from our data whether this was the case.

Taken together, these methodological issues likely resulted in some underestimates of the examined outcomes. However, the aims of the study were not to obtain the population prevalence estimates^53^ but to explore putative differences in self-reported mental and physical health-among Norwegian adolescents associated with the COVID-19 period and conditions. To our knowledge, this is the first study to do so, while using a prospective nationwide sample and internationally validated measures (ie, PHQ-9) and controlling for multiple covariates, including prior levels of all examined outcomes. In that regard, this study substantively extends on other Norwegian reports of adolescent well-being during the pandemic.^4,5,28^

## Conclusions

This cohort study found that Norwegian adolescents who started high school during the first year of the COVID-19 pandemic were significantly less likely to participate in organized sports than their sociodemographically equivalent peers from before the pandemic but were similar in terms of the depression symptoms, friendships, and physical health. However, adolescents reporting HPA were significantly more likely to report worse mental and physical health than their pre–COVID-19 peers. Continued monitoring and examination of longer temporal trends are essential, as is the inclusion of additional sociodemographic groups and geographic regions.

Additional research is needed to identify risk factors and characteristics associated with heightened stress and anxiety during the pandemic period among adolescents. Public health strategies cannot proceed without identifying adolescents who are worried about infection, school, and other health and social consequences of the pandemic and without understanding whether those worries are perceived or genuine (and if so, why) and what other risk factors may be associated with them. Such at-risk groups of adolescents may be disproportionately affected by the pandemic and may require specialized strategies addressing their mental and physical health needs.^5^
